# Neuroserpin normalization by mesenchymal stem cell therapy after encephalopathy of prematurity in neonatal rats

**DOI:** 10.1038/s41390-024-03412-z

**Published:** 2024-08-01

**Authors:** Lan-Wan Wang, Chien-Wei Hsiung, Ching-Ping Chang, Mao-Tsun Lin, Shyi-Jou Chen

**Affiliations:** 1 Department of Pediatrics, Chi Mei Medical Center, Tainan, Taiwan, ROC; 2 Department of Biotechnology and Food Technology, Southern Taiwan University of Science and Technology, Tainan, Taiwan, ROC; 3 School of Medicine, National Sun Yat-sen University, Kaohsiung, Taiwan, ROC; 4 Department of Medical Research, Chi Mei Medical Center, Tainan, Taiwan, ROC; 5 Department of Environmental and Occupational Health, College of Medicine, National Cheng Kung University, Tainan, Taiwan, ROC; 6 Department of Pediatrics, Tri-service General Hospital, School of Medicine, National Defense Medical Center, Taipei, Taiwan, ROC

## Abstract

**Background:**

Hypoxic-ischemia (HI), infection/inflammation and reperfusion injury are pathogenic factors of encephalopathy of prematurity, which involves maturational/neurotrophic disturbances in oligodendrocyte progenitor cells (OPC) and neurons/axons. Mesenchymal stem cells (MSCs) might facilitate neuroserpin production, which is neurotrophic for OPC/neurons. This study investigated MSC effects on developmental disturbances after lipopolysaccharide (LPS)-sensitized HI/reperfusion (LHIR) injury and the relation to neuroserpin expression.

**Methods:**

Postnatal day 2 (P2) rat pups received intraperitoneal LPS (5 µg/kg) injection followed by HI (unilateral common-carotid-artery ligation and 6.5% oxygen exposure for 90 min) and post-HI reperfusion (release of ligation). MSCs (5 × 10^4^ cells) were injected into the left lateral ventricle at 24 h post-LHIR. Neurological tests and brain tissue examinations were performed between P5 and P56.

**Results:**

After LHIR injury, MSC therapy significantly reduced cell death in subplate neurons, attenuated axonal damage, and facilitated synaptophysin synthesis in the cortex. It also alleviated OPC maturation arrest and preserved the complexity of myelinated axons in the white matter, leading to cognitive, motor and behavioral functional improvements. These beneficial effects were linked to restored neuroserpin expression in subplate neurons.

**Conclusions:**

MSC therapy ameliorated developmental disturbances after LHIR injury through protection of neuroserpin expression, serving as a promising approach for treating encephalopathy of prematurity.

**Impact:**

- Neuroserpin is secreted by subplate neurons and may regulate the development of neurons and oligodendrocyte-axon contact for myelination in the premature brain.
- LPS-sensitized hypoxic-ischemia/reperfusion (LHIR) injury caused the developmental disturbances of neurons/axons and oligodendrocytes, and lowered neuroserpin levels in a neonatal rat model simulating encephalopathy of prematurity.
- Mesenchymal stem cell therapy alleviated the developmental disturbances after LHIR injury through protection of neuroserpin expression in subplate neurons, offering a new perspective on potential treatment for encephalopathy of prematurity.

## Introduction

Diffuse white matter injury is the most common form of encephalopathy of prematurity (EoP), leading to neurological deficits such as cognitive impairment, cerebral palsy, or autism spectrum disorder in extremely preterm (<28 weeks of gestation) survivors.^1–3^ Hypoxic ischemia (HI) and infection/inflammation are the two major pathogenic factors of EoP, and may aggravate brain injury through inflammation-sensitized HI, or exert cumulative effects to increase the risk of neurodevelopmental impairment.^1,4,5^ In addition, post-HI reperfusion with high oxygen concentration during resuscitation may trigger reactive oxygen/nitrogen species production to further damage the brain.^1,6^

White matter injury in preterm infants is often accompanied by neuronal/axonal diseases with reduction of regional brain volumes detected on magnetic resonance imaging.^7,8^ Experimental evidence also suggests that EoP may be a complex amalgam of primary destructive diseases and secondary developmental disturbances (or brain dysmaturation) involving pre-myelinating oligodendrocytes (pre-OLs), subplate neurons and axons.^7,8^ Pre-OLs are the target cells of damage in the white matter,^1,9^ while subplate neurons in the subcortical white matter play pivotal roles in cortical layer formation, axonal outgrowth, synaptic connections, and myelination in early brain development.^10,11^ Several experimental studies have demonstrated destructive contributors such as neuroinflammation and neurovascular damage in the premature brain,^12–14^ but few have investigated the developmental disturbances,^15,16^ which are potentially related to an insufficiency of neurotrophic factors.^7^

Neuroserpin is a serine protease inhibitor secreted by cortical plate and subplate neurons during early brain development, with its distribution concentrated in the subcortical area between 23 and 32 weeks of gestation,^17^ which fall within the developmental window of vulnerability for EoP.^9^ Neuroserpin plays key roles in not only neuronal differentiation, axonogenesis and synaptic refinement,^18,19^ but also oligodendrocyte-axon interactions for myelination through up-regulation of cell adhesion molecules such as N-cadherin.^20–22^ In addition to its neurotrophic roles, neuroserpin has shown protective effects against neuroinflammation and ischemia/reperfusion-induced injury in experimental studies.^23–25^ Very few studies have investigated the effects of neuroserpin on neonatal brain injury,^26^ and it remains unclear whether neuroserpin helps ameliorate developmental disturbances in the immature brain after inflammation-sensitized HI/reperfusion injury.

Displaying anti-inflammatory properties as well as providing trophic support with low immunogenicity, mesenchymal stem cells (MSCs) transplantation has emerged as a promising therapy for many neurological disorders,^27^ and was reported to be effective in animal models of HI and/or inflammation-induced neonatal brain injury.^15,16,28,29^ MSCs may facilitate neurotrophic factor production through paracrine secretion or up-regulation of endogenous synthesis.^27,30^ Using an animal model of EoP induced by low-dose lipopolysaccharide (LPS) and subthreshold HI/reperfusion in postnatal day 2 (P2) rat pups (brain maturation equivalent to 23–25 weeks’ gestation in human), this study tested the hypothesis that MSC therapy may alleviate the developmental disturbances after LPS-sensitized HI/reperfusion brain injury to improve neurocognitive, motor and behavioral function through protecting neuroserpin/N-cadherin signaling.

## Methods

### Animals

Pregnant Sprague-Dawley rats (12–14 days of gestation) were purchased from BioLASCO Co., Ltd Taiwan (Taipei, Taiwan) and housed under standard housing conditions with a 12-h light/dark cycle. After delivery, rat pups were cared for by their mothers. The Institutional Animal Care and Use Committee in Chi Mei Medical Center approved the study protocols, and the reports were written according to the ARRIVE checklist. A total of 120 pups were utilized in the study, and the sample size for each experiment was determined using GPower 3.1 software. During the experiments, rat pups that appeared cyanotic and apneic were removed immediately and provided with fresh air. If the pups did not recover and remained comatose for more than 3 h after the intervention, they were humanely euthanized via cervical dislocation. To reduce the impact of individual variations on the data, pups were eliminated from the study if their experiments had been interrupted. The elimination rate in this study was 15%.

### A neonatal rat model of EoP induced by LPS-sensitized HI/reperfusion

The neonatal rat model of EoP employed in this study was adapted from our previously established protocols.^4,12^ P2 rat pups received an intraperitoneal injection of 5 µg/kg LPS (Escherichia coli O55:B5; L2880, Sigma-Aldrich, St Louis, MO) 3 h before HI. To prevent LPS-induced fluctuations in body temperature, the pups were returned to their dams and kept in an incubator, maintaining a stable temperature of 33–34 °C until HI induction. The HI model involved unilateral ligation of the right common carotid artery, followed by a hypoxic challenge. Under anesthesia with isoflurane (3–5%), pups received an incision on the neck to expose the right common carotid artery, and a temporary ligature was applied using 4-0 surgical silk. After surgery, the pups were allowed to recover for one hour in an incubator. Subsequently, they were placed in a hypoxia chamber maintained at 36–36.5 °C, with humidified 6.5% oxygen flowing at 3 L/min for 90 min. Following the hypoxic period, the ligature was removed, and the artery was gently compressed with forceps to facilitate post-HI reperfusion. The neck wound was sutured, and the pups were returned to their dam for recovery. In this model, rat pups undertaking temporary ligation had more neuroinflammation and brain injury than those receiving permanent ligation (Supplementary Fig. 1), suggesting the additional impact of post-HI reperfusion. The mortality rate in the neonatal rat model was ~10–12%.

### Human mesenchymal stem cell culture

Commercially available human MSCs were purchased from Merck (# SCC034, Billerica, MA). The cells were cultured in low-glucose DMEM (#31600034, Gibco, Waltham, MA) with 10% FBS, 0.5% human fibroblast growth factor-basic (#GF003, Millipore, St Louis, MO) and 2 mM L-glutamine at 37 °C. MSCs were identified as CD90+/CD73+/CD105+ and CD34−/CD45− cells using FACS flow cytometry (Beckman Coulter, Indianapolis, IN) and monoclonal antibodies, and further verified by their ability of differentiation into the osteogenic, adipogenic or chondrogenic lineage, as described in our previous study (Supplementary Fig. 2).^31^

### Experimental groups

The P2 rat pups were randomly assigned to three groups—sham control (without LPS, HI or reperfusion), LPS-sensitized HI with reperfusion (LHIR), and LHIR followed by MSC treatment (LHIR + MSC) at 24 h post-insult (P3). The MSCs (5 × 10^4^ cells/5 μl) were intracerebrally injected into the left lateral ventricle under anesthesia according to the published method.^32^ Male and female pups were randomly allocated in different experimental groups. Rats in each group received sequential tests from P5 to P56 for performance of neurological function, and were sacrificed for brain tissue examinations on P5, P10, P16, and P23 (Supplementary Fig. 3).

### Immunofluorescence staining

The rat brains were paraffinized and coronally sectioned (10-μm thick) from the genu of the corpus callosum to the end of the dorsal hippocampus. Four coronal sections, two at the level of the striatum (0.26 mm and 0.92 mm posterior to the bregma) and another two at the levels of the dorsal hippocampus (3.14 mm and 4.16 mm posterior to the bregma), were selected according to a rodent brain atlas and assessed for each brain.^33^

After blocking, the sections were incubated overnight at 4 °C with one or two of the following primary antibodies. Neuronal markers included anti-complexin 3 (subplate neuron marker; 1:200, #122302, Synaptic Systems, Göttingen, Germany), anti-doublecortin (1:200, #ab153668, abcam, Boston, MA), anti-amyloid precursor protein (axonal injury marker; 1:200, #ab32136, abcam), anti-synaptophysin (1:200, #ab32127, abcam), and anti-neuroserpin (1:200, #ab33077, abcam); while oligodendroglial markers comprised anti-A2B5 (1:100, #ab53521, abcam), anti-O4 (1:500, #MAB1326, R&D, Minneapolis, MN), and anti-myelin basic protein (MBP; 1:200, #MAB386, Sigma-Aldrich, Temecula, CA). Other markers included anti-ionized calcium binding adaptor molecule 1 (Iba-1; 1: 500, #019-19741, Wako, Osaka, Japan) and anti-TNF-α (1:200, #ab307164, abcam). The sections were then washed and incubated with Alexa Fluor 594 or 488 secondary antibodies (1:200; Invitrogen, Waltham, MA). The slides were photographed for red (Alexa Fluor 594) and green (Alexa Fluor 488) fluorescence with a fluorescent microscope.

The quantification of fluorescence image areas was conducted using an image software (Image-Pro Plus 6.0; Media Cybernetics, Bethesda, MD). Measurements were performed at 100× (0.579 mm^2^) or 200× (0.145 mm^2^) magnification per visual field. Three visual fields in the medial, middle and lateral areas of each hemisphere per section and four sections per brain were analyzed.^12^ The ratios of the antigen-positive area to the cross-sectional area were averaged and compared in the same hemispheres between groups.

### Coherency of myelinated axons

Coherency of myelinated axons, as an inverse measure of complexity of white matter microstructure, was assessed using the “OrientationJ measure” function of ImageJ plugin OrientationJ in the selected whole micrograph.^34^

### Western blot analysis

The brain tissue was homogenized in cold lysis buffer and the protein concentrations were determined using a Bio-Rad Protein Assay kit (Bio-Rad Laboratories, Hercules, CA). Samples (50 μg) were separated using 10% SDS-PAGE and blotted onto polyvinylidene fluoride membranes. After blocking, the membranes were incubated with primary antibodies at 4 °C overnight. Immunoreactivity was detected by horseradish-conjugated secondary antibodies and visualized by enhanced chemiluminescence. The primary antibodies used were anti-neuroserpin (1:1000, #ab269277, abcam), anti-N-cadherin (1:1000, #ab18203, abcam), and anti-β-actin (1:5000; Invitrogen). The band signals were quantified using an imaging software (ImagePro Plus 6.0; Media Cybernetics, Bethesda, MD).

### Neurobehavioral, neurocognitive, and neuromotor function assessment

#### Surface righting reflex

P5 pups were placed on their backs, and the time spent for turnover was recorded.^35^

#### Negative geotaxis

P9 pups were placed on a 15° inclined surface and facing toward the bottom of a slope. The pups would turn around and start to climb the slope, and the time spent for completing the 180° turnaround was recorded.^35^

#### Wire hanging test

P10 pups were placed on an iron wire net. The net was then turned upside down so that the pups hung on the net. The time from suspension to fall was recorded.^35^

#### Pouncing and pinning (social play behavior)

P28 rats were habituated to the test chamber for 10 min on 2 consecutive days. On the third day, the rats were secluded for 2.5 h and put into the test chamber together with nonfamiliar, weight- and sex-matched rats of the same experimental group. For 15 min, social play behavior was videotaped for pouncing (trying to rub the nape of other rats with the nose) and pinning (standing on another rat while the back of the other rat is lying on the floor) using a video recording device.^14^

#### Repetitive self-grooming (repetitive behavior)

 On P35, rats were placed in a transparent cage containing only fresh bedding material for 20 min. The time spent grooming the hair was recorded using a video recording device.^14^

#### Rotarod test (neuromotor function)

The rotarod consists of a circular rod turning at a constant or increasing speed. P35 rats were placed on the rotating rod, and the time and the maximal turning speed staying on the rod were recorded.

#### Open field test (exploratory behavior)

The P49 rats were placed in a circular field with a diameter of 1 m for 5 min, and software was used to track their motor behavior automatically. The circular field was divided into two parts: the outer edge (12.5 cm) and the inner area (75 cm in diameter). The number of times that animals left the outer zone (sheltered by the walls of the arena) and entered the inner zone within 5 min was recorded.

#### Passive avoidance task (learning and memory)

The latency to refrain from crossing into the punished compartment served as an index of the ability to avoid and allowed memory to be assessed in P56 rats. The apparatus (Shuttlebox-Passive Avoidance, Accuscan Instrument, Inc., Columbus, OH) consisted of a rectangular chamber divided into two compartments by an automatic guillotine door. An overhead stimulus light illuminated one compartment, and the other was black. A rat was placed in the illuminated chamber during the pre-training trial. Once the rat had completely entered the dark compartment, the door was closed and a mild electric shock was applied to the floor grid for 3 s. A retention test was conducted 48 h after the pre-training trial to assess the retention of passive avoidance performance. The rat was placed back in the lighted chamber, and the time it took to enter the dark room with four paws was measured.

### Statistical analysis

Statistical analyses were performed using GraphPad Prism 8 software (GraphPad Software Inc., CA). Data are expressed as the mean ± standard deviation (SD). The immunofluorescence staining data with non-normal distribution were analyzed using the Kruskal-Wallis test and Dunn’s post-hoc test. One-way analysis of variance with Tukey’s post hoc test was used to analyze the Western blotting and behavioral performance. *P* values < 0.05 were considered statistically significant. No outliers were excluded from the statistical analysis. The person charged with outcome measurements of staining, Western blotting, or neurological function was blind to treatments.

## Results

### MSC therapy attenuated neuroinflammation, neuronal death, axonal damage, and synaptic disruption after LPS-sensitized HI/reperfusion (LHIR) injury

**Fig. 1:**
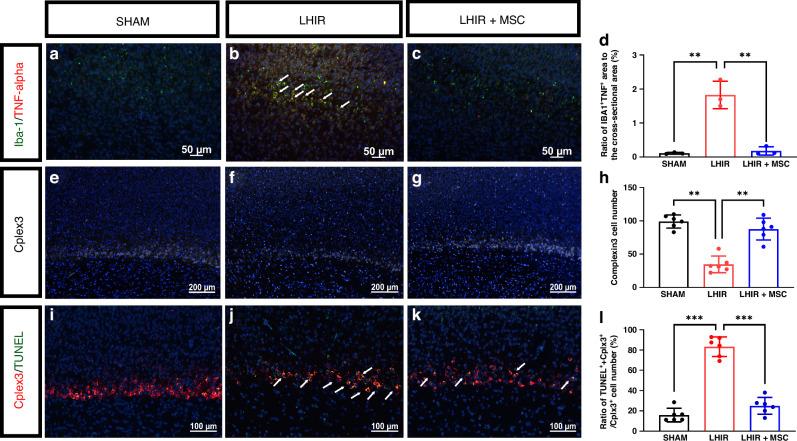
MSC treatment reduced neuroinflammation and subplate neuron cell death on P5 after LHIR injury. **a**–**d** Double immunofluorescent staining showed increases of Iba-1-positive microglia with TNF-α co-expression (white arrows) in the LHIR group compared to the sham and LHIR + MSC groups. **e**–**h** There was significantly decreased cell numbers of complexin-3 (Cplex3)-positive subplate neurons in the LHIR group compared to the other two groups. **i**–**l** TUNEL stain for subplate neurons showed significantly increased numbers of cells with co-localization (white arrows) in the LHIR group compared to the LHIR + MSC group. *n* = 6 in each group. Values are mean ± SD. **p < 0.01.

At post-insult 72 h (P5), the LHIR group showed increased activation of microglia with elevated pro-inflammatory cytokine TNF-α expression, whereas MSC treatment significantly attenuated neuroinflammation (Fig. 1a–d, *p* < 0.01). Compared to the sham group, there was significant decreases in the numbers of complexin-3-positive subplate neurons (Fig. 1e–h) with increased cell death (Fig. 1i–l) in the LHIR group. MSC treatment reduced cell death and preserved most subplate neurons (Fig. 1e–l, all *p* < 0.01).

**Fig. 2:**
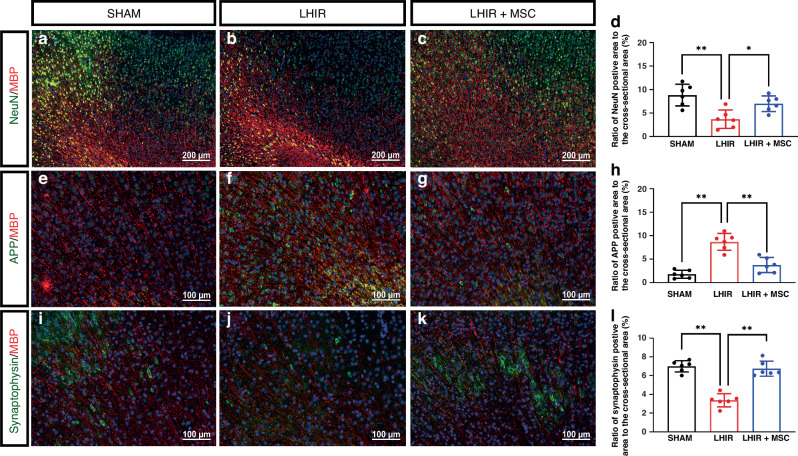
MSC treatment increased mature neuron numbers, attenuated axonal injury, and restored synaptic formation on P23 after LHIR injury. **a**–**d** Immunofluorescent staining showed the LHIR group had significantly decreased numbers of NeuN-positive neurons compared to the sham and LHIR + MSC groups. **e**–**h** There was significantly increased expression of amyloid precursor protein (APP) in the LHIR group than in the other two groups, indicating axonal injury. **i**–**l** Synaptophysin expression was reduced in the LHIR group but was restored in the LHIR + MSC group. *n* = 6 in each group. Values are mean ± SD. *p < 0.05; **p < 0.01.

In the neonatal rodent brain, the maturation of cortical plate neurons is completed by P7, followed by axonal outgrowth and synaptic formation reaching a mature morphology at the end of the first month.^36^ On P23, we observed significant decreases in NeuN-positive mature neurons in both the ipsilateral (Fig. 2a–d, *p* < 0.01) and contralateral (Supplementary Fig. 4a–d, *p* < 0.01) cortices in the LHIR group. In contrast, the LHIR + MSC and sham groups did not show differences in cell numbers. The LHIR group also displayed axonal injury with significant accumulation of amyloid precursor protein in axons (Fig. 2e–h), and reduced synaptic formation with lower synaptophysin expression (Fig. 2i–l) compared to the sham group (all *p* < 0.01); whereas MSC treatment ameliorated axonal injury and restored synaptic connections (Fig. 2e–l, *p* < 0.01).

### MSC therapy alleviated the maturation arrest of oligodendrocyte progenitors and maintained the complexity of myelinated axons after LHIR injury

**Fig. 3:**
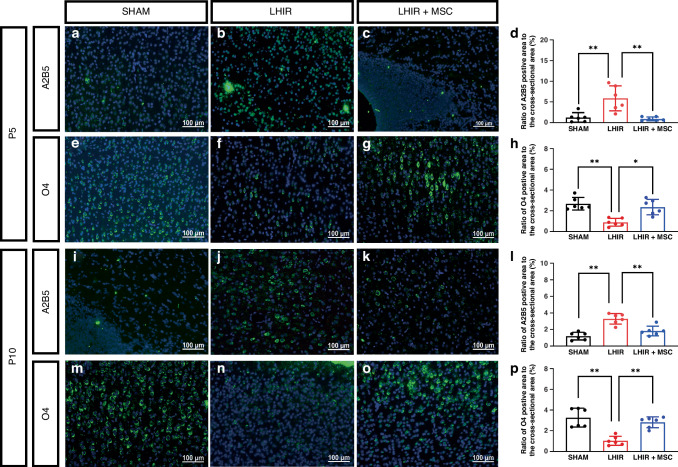
MSC treatment ameliorated maturation arrest of oligodendrocyte progenitors after LHIR injury. Immunofluorescent staining on P5 and P10 showed the LHIR group had significantly increased A2B5-positive (**a**–**d, i**–**l**) and decreased O4-positive cells (**e**–**h**, **m**–**p**) compared to the sham and LHIR + MSC groups, while the latter two groups had no substantial differences. n = 6 in each group. Values are mean ± SD. *p < 0.05; **p < 0.01.

**Fig. 4:**
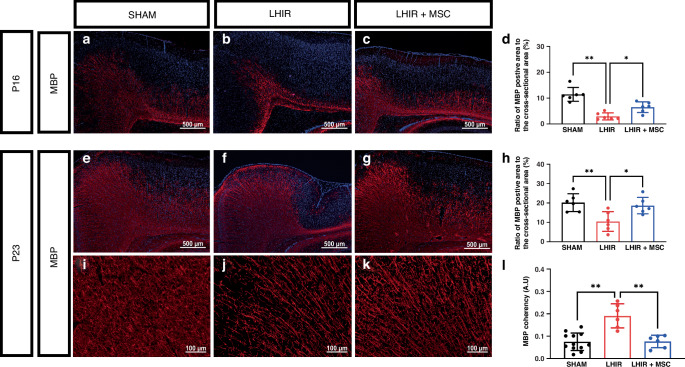
MSC treatment alleviated hypomyelination and maintained the complexity of myelinated axons after LHIR injury. Immunofluorescent staining on P16 (**a**–**d**) and P23 (**e**–**h**) showed significantly reduced expression of myelin basic protein (MBP) in the LHIR group. The LHIR + MSC and sham groups did not differ in the expression. *n* = 6 in each group. **i**–**l** The LHIR group (n = 6) had significantly increased coherency of myelinated axons on P23 compared to the sham (*n* = 12) and LHIR + MSC (*n* = 6) groups, while the latter two groups had comparable axonal coherency. Values are mean ± SD. *p < 0.05; **p < 0.01.

During the lineage progression of rodent oligodendrocyte, A2B5-positive oligodendrocyte precursor cells (OPCs) appear first in the embryonic stage and gradually decline after birth, followed by O4-positive pre-OLs from P2 onward, and finally MBP-positive mature oligodendrocytes predominate from P14.^9,37^ At post-insult 72 h (P5) and 8 days (P10) in this study, the LHIR group had significantly increased A2B5-positive and decreased O4-positive cells compared to the sham group (*p* < 0.01), while MSC treatment normalized the developmental sequence of oligodendrocyte lineage (Fig. 3a–h on P5; 3i-p on P10). On P16 and P23, significant hypomyelination with decreased MBP expression occurred in the LHIR group (*p* < 0.01), whereas MSC treatment preserved MBP expression (Fig. 4a–d on P16, e-h on P23). On P23, the LHIR group also displayed increased coherency of myelinated axons in the ipsilateral (Fig. 4i–l, *p* < 0.01) and contralateral (Supplementary Fig. 4e–h, *p* < 0.01) hemispheres, indicating reduced complexity with less axonal outgrowth and arborization. The LHIR + MSC group restored axonal complexity to levels similar to those in the sham group.

### MSC therapy improved neurofunctional impairment after LHIR injury

**Fig. 5:**
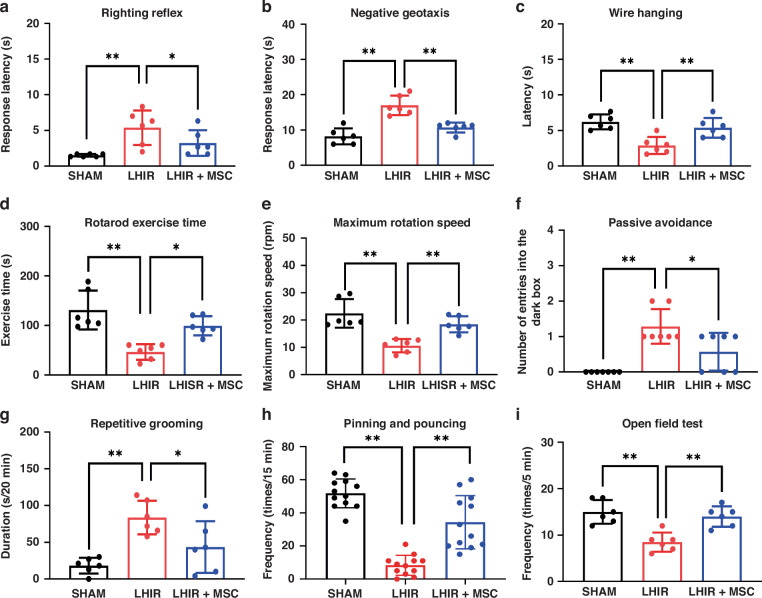
MSC treatment improved neurological function after LHIR injury. Compared to the sham group, rat pups in the LHIR group had significantly worse performance in the innate behavior (**a**–**c**), neuromotor (**d, e**) and learning/memory (**f**) function, and exhibited more autistic-like behavior (**g**–**i**). The LHIR + MSC and the sham groups had comparable performance in the neurological **(a**–**f)** and behavioral (**g**–**i**) tests. *n* = 6 per group in (**a**–**c**), conducted between P5 and P10; *n* = 8 per group in (**d**–**i**), conducted between P28 and P56. Values are mean ± SD. *p < 0.05; **p < 0.01.

In the early postnatal period (P5-P10), rat pups in the LHIR group spent more time on righting reflex and negative geotaxis, and had less hanging time in wire hanging test, while pups in the LHIR + MSC group had significantly better performance (Fig. 5a–c, all *p* < 0.05). After the first month of life (P28-P56), rats in the LHIR + MSC group had better neuromotor and cognitive function than those in the LHIR group, with longer time walking on the rolling wheel and faster rotating speeds in the Rotarod test, and lower frequencies of erroneous entry into the dark compartment in the passive avoidance task (Fig. 5d–f, all *p* < 0.05). Rats in the LHIR group exhibited autistic-like behavior including a longer duration of repetitive self-grooming, reduced pinning/pouncing for social interactions, and a lower frequency of exploratory behavior in the open field test (Fig. 5g–i, all *p* < 0.01); while rats in the LHIR + MSC and the sham control groups had comparable performance in behavior. There were no sexual differences in the performance of open field test and passive avoidance task, which were performed between P49 and P56 at the ages corresponding to sexual maturity (Supplementary Fig. 5).

### MSC treatment restored neuroserpin/N-cadherin expression in neuronal cells after LHIR injury

**Fig. 6:**
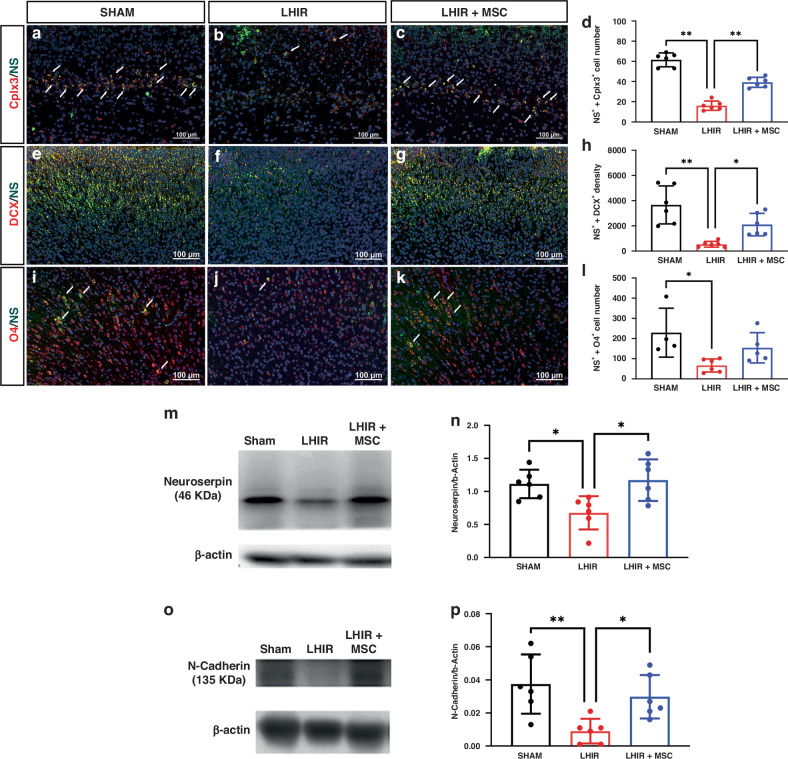
MSC treatment restored neuroserpin expression in neuronal cells after LHIR injury. Double immunofluorescent staining on P5 showed the LHIR group had significantly decreased numbers of complexin-3 (Cplx3)-positive subplate neurons (**a**–**d**) and doublecortin (DCX)-positive neurons (**e**–**h**) with neuroserpin (NS) expression, while the LHIR + MSC and sham groups did not differ in numbers of neuronal cells with co-expression (white arrows). There were few O4-positive oligodendrocyte progenitor cells with co-expression of neuroserpin (white arrows) in each group, especially in the LHIR group (**i**–**l**). Western blotting in the cortex showed the LHIR group had significantly reduced expressions of neuroserpin (**m**, **n**) and N-cadherin (**o**, **p**), which were restored in the LHIR + MSC group (**m**–**p**). *n* = 6 in each group. Values are mean ± SD. *p < 0.05; **p < 0.01.

We further examined the potential roles of neuroserpin in the protective mechanisms of MSC therapy. In P5 sham control rats, both subplate neurons (positive for complexin-3) and neuron progenitor cells (positive for doublecortin) displayed neuroserpin expression (Fig. 6a, e). After LHIR injury, there were significantly decreased numbers of neuronal cells with neuroserpin expression, which were restored after MSC treatment (Fig. 6b–d, f–h, all *p* < 0.05). There were few O4-positive oligodendrocytes with neuroserpin expression in each group, especially in the LHIR group (*p* < 0.05); while the LHIR + MSC and the sham groups had similar cell numbers of neuroserpin-expressing oligodendrocytes (Fig. 6i–l). Western blotting further demonstrated that MSC therapy restored not only neuroserpin but also N-cadherin expression in the cortex after LHIR injury (Fig. 6m–p, all *p* < 0.05), implicating a potential neuroserpin/N-cadherin signaling pathway.

## Discussion

Using a neonatal rat model to simulate EoP, this study investigated the therapeutic effects of MSC transplantation on the developmental disturbances of neurons/axons and oligodendrocyte in relation to neuroserpin expression. Following an injury induced by LPS-sensitized HI/reperfusion, we found microglial activation followed by increased cell death in subplate neurons, reduced mature neurons, axonal destruction, and decreased synaptic formation. The damage to neurons/axons might lower neuroserpin levels in the cortex, along with arrested maturation of oligodendrocyte progenitors leading to hypomyelination in the white matter. The maturational disturbances during early brain development resulted in not only cognitive and neuromotor functional impairments but also autistic-like behaviors, reflecting neurodevelopmental disorders seen in children born extremely preterm. MSC treatment mitigated neuronal death, protected against injury in both the cortex and white matter, improved neurological and behavioral outcomes, and elevated neuroserpin and N-cadherin levels in neurons. These findings suggest that neuroserpin/N-cadherin signaling might play critical roles in the reparative mechanisms of MSC therapy (Supplementary Fig. 6).

The pathogenic mechanism of EoP is a combination of a destructive injury and the subsequent trophic/maturational (i.e., developmental) disturbances.^7,8^ Most experimental studies of preterm brain injury focused on the destructive component, which was induced by exposure to HI, or to neurotoxin such as LPS or pro-inflammatory cytokines in neonatal animals.^38–41^ Our previous studies have found that subthreshold HI was sensitized by low-dose LPS to trigger significant injuries in the neonatal rat brain, characterized by microglia-mediated neuroinflammation and neurovascular damage.^12,13^ This study further explored the developmental disturbances and neurobehavioral outcomes using the modified model of LPS-sensitized HI brain injury followed by post-HI reperfusion, which occurs frequently in preterm neonates but is rarely considered in previous studies.

Subplate neurons reach their culmination size during the peak period for occurrence of EoP (23–32 weeks of gestation), and serve as a platform for axonal guidance and synaptic contacts linking the development of cerebral cortex, subcortical white matter and deep nuclei.^10,11,42^ An animal study has shown that circuit changes in the subplate neurons after HI in P1 rat pups led to altered brain development.^43^ Our study found increased cell death with decreased numbers of subplate neurons at 72 h (P5) after LHIR injury. Damage to the subplate neurons potentially induces degeneration of axons and their originating neurons through retrograde or anterograde effects, and axonal degeneration further results in hypomyelination.^7,44,45^ Our findings provided evidence of neuronal degeneration, axonal injury, and impaired synaptic formation at post-insult 21 days (P23).

During the lineage progression of oligodendrocytes, O4-positive pre-OLs predominate in the P2 rodent brain, which coincides with the developmental window of vulnerability for EoP in the human brain.^37,46^ Pre-OLs contact axons extending to and from the hub of subplate neurons for myelination, and are selectively vulnerable to HI and reperfusion-induced oxidative stress.^47,48^ The damaged pre-OLs are shown to undergo cell death or maturation arrest leading to hypomyelination,^49–51^ or trigger axonal degeneration leading to impaired neuronal development.^7,45,52^ After LHIR on P2, we found increased A2B5-positive OPCs and decreased O4-positive pre-OLs on P5, followed by diminished MBP expression and reduced complexity of myelinated axons on P23. Our findings implicated that hypomyelination was attributed to axonal injury and early maturation arrest with failure of OPC differentiation into pre-OLs.

This study verified the hypothesis that MSC therapy ameliorated the developmental disturbances and improved neurobehavioral outcomes after LHIR injury through protection of neuroserpin/N-cadherin signaling in subplate neurons and neuronal progenitor cells. Neuroserpin is expressed in the neocortex, hippocampus and limbic system that are responsible for learning, memory and social behavior, and reaches a peak level in the rodent brain during the first week of life.^53,54^ Although neuroserpin is an inhibitor of tissue plasminogen activators, it is shown in animal studies to attenuate destructive brain injury such as ischemic stroke via non-enzymatic effects.^18,25^ Experimental studies also implicate neuroserpin is involved in regulating critical cellular functions through crosstalk with downstream signaling networks.^18,22^ During the process of neural circuit formation that occurred in the platform of subplate neurons, neuroserpin might not only exert anti-apoptotic effects on neural progenitor cells, but also promote axonal outgrowth, synaptic connections, and oligodendrocyte-axon contact for myelination through regulation of cell adhesion molecules such as N-cadherin.^18–22^ Lack or dysregulation of neuroserpin leads to neuropsychiatric disorders such as cognitive impairment, social ability deficits, and defective exploratory behavior.^18,53,55^ Despite restoring neuroserpin expression correlates with improved brain histology and neurobehavioral outcomes in this study, it does not establish a direct causal relationship, and how MSC therapy normalized neuroserpin expression after LHIR injury remains unclear. Further studies are needed to elucidate the underlying mechanisms and the causative links between neuroserpin expression and brain repair.

There are limitations in this study. First, this study used an intracerebroventricular route for MSC transplantation, which is not feasible in clinical practice for infants born very preterm, despite the superior therapeutic efficacy provided by targeted delivery and better paracrine potency of MSCs compared to other routes.^56,57^ Our findings of intracerebral MSC effects might also be observed with systemic intravenous administration, which has shown therapeutic effects comparable to those via the intracerebroventricular route in animal studies of preterm brain injury.^58,59^ Since the paracrine action, rather than direct regeneration, is regarded as the primary protective mechanism of MSC transplantation, strategies to enhance the paracrine potency, such as preconditioning or genetic engineering of MSCs, may improve the therapeutic efficacy.^59^ Second, the MSC dose used in this study has limited clinical translatability. The determination of the MSC dose in this study was modified from a previous study of intraventricular hemorrhage (IVH) in P5 Sprague–Dawley rat, which showed that injecting 1 × 10^5^ MSCs into the lateral ventricles on P6 improved severe IVH.^60^ Considering the treatment time point in our study is on P3 when the ventricular size is smaller than that on P6, we administered half the dose used in that study (5 × 10^4^ MSCs) for treatment. The optimal dose of MSC transplantation needs to be determined according to the specific brain injury site and brain disorders with the specific source, timing and route of administration in different species,^59^ especially safety in the clinical context. Third, pharmacological approaches or genetically modified mice might be used to verify the potential mechanisms of MSC effects through neuroserpin/N-cadherin signaling. Fourth, although sex-related differences are largely attributed to hormonal variations after sexual maturity (~40–60 days of age in rats),^61^ there is growing evidence suggesting that sexual differences should be considered in neonatal rodent models during the first month of life.^62^ Despite no observed differences in brain damage and outcome performance between male and female rats in this study, we cannot completely exclude the possibility of sex-related differences. Future studies may investigate the potential effects of sex on the underlying mechanisms of EoP.

In conclusion, using a novel model of LPS-sensitized HI/reperfusion injury based on the pathogenesis of EoP, this animal study demonstrated the developmental disturbances in neurons and oligodendrocytes and the subsequent impairment of neurobehavioral functions, and uncovered the roles of neuroserpin in brain repair mechanisms of MSC therapy. Future research may investigate the therapeutic potential of MSC-conditioned medium or MSC-derived extracellular vesicles/exosomes for better clinical application, and explore other reparative mechanisms of MSC secretome for developmental disturbances in the premature brain.

## Supplementary information

Supplementary Material

## Data Availability

The data that support the findings of this study are available from the corresponding author upon reasonable request.
