# Correction: Neuroserpin normalization by mesenchymal stem cell therapy after encephalopathy of prematurity in neonatal rats

**DOI:** 10.1038/s41390-025-03889-2

**Published:** 2025-03-20

**Authors:** Lan-Wan Wang, Chien-Wei Hsiung, Ching-Ping Chang, Mao-Tsun Lin, Shyi-Jou Chen

**Affiliations:** 1 Department of Pediatrics, Chi Mei Medical Center, Tainan, Taiwan, ROC; 2 Department of Biotechnology and Food Technology, Southern Taiwan University of Science and Technology, Tainan, Taiwan, ROC; 3 School of Medicine, National Sun Yat-sen University, Kaohsiung, Taiwan, ROC; 4 Department of Medical Research, Chi Mei Medical Center, Tainan, Taiwan, ROC; 5 Department of Environmental and Occupational Health, College of Medicine, National Cheng Kung University, Tainan, Taiwan, ROC; 6 Department of Pediatrics, Tri-service General Hospital, School of Medicine, National Defense Medical Center, Taipei, Taiwan, ROC

Correction to: *Pediatric Research* 10.1038/s41390-024-03412-z, published online 01 August 2024

In this article the affiliation details for Author Shyi-Jou Chen were incorrectly given as ‘Department of Pediatrics, School of Medicine, National Defense Medical Center, Taipei, Taiwan, ROC.’ but should have been ‘Department of Pediatrics, Tri-service General Hospital, School of Medicine, National Defense Medical Center, Taipei, Taiwan, ROC’.

The original article has been corrected.

