# Supplementary material for: Neuroserpin normalization by mesenchymal stem cell therapy after encephalopathy of prematurity in neonatal rats
Source: Pediatr Res. 2024 Aug 1;97(3):1199–208. doi: 10.1038/s41390-024-03412-z (PMC12055564; doi:10.1038/s41390-024-03412-z)
Supplement: Supplementary file 1 — Supplementary Material [file 41390_2024_3412_MOESM1_ESM.pdf]

Supplementary Fig. 1

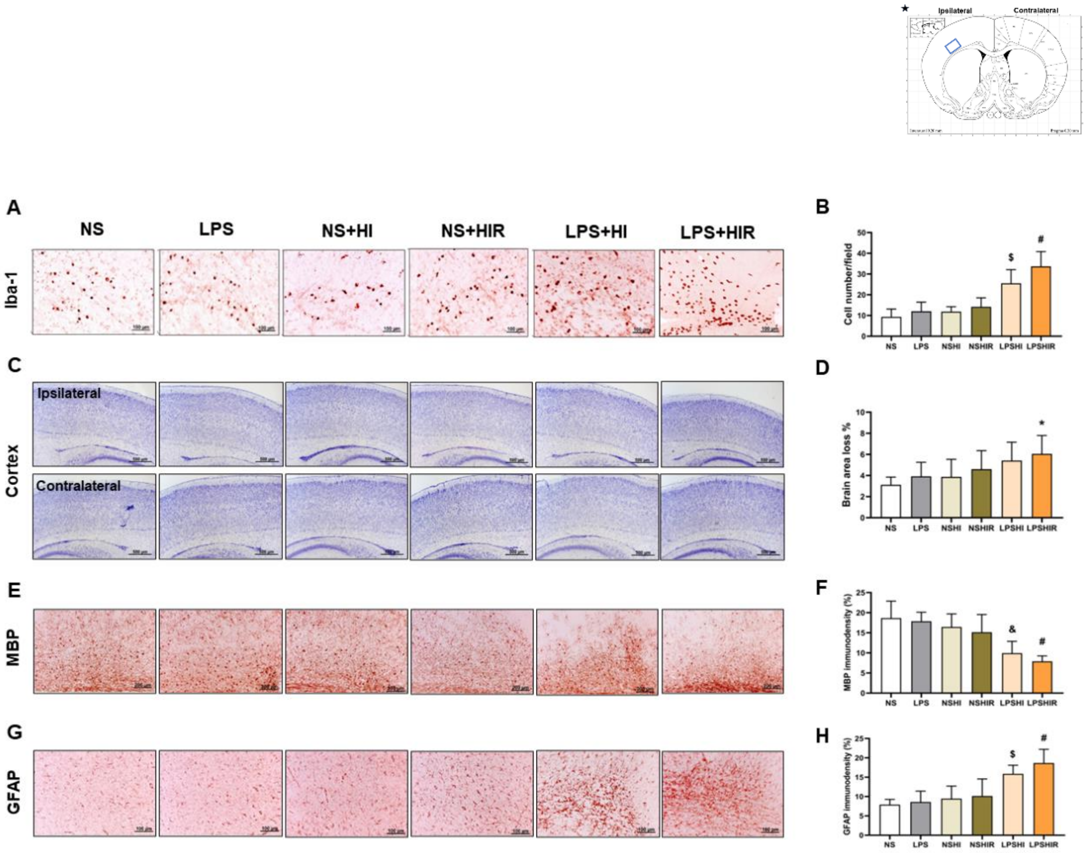

Legend. Comparison of neuroinflammation and brain injury among normal saline (NS)-injected control, lipopolysaccharide (LPS)-injected, NS injection followed by hypoxic-ischemia (NS+HI), NS injection followed by HI/reperfusion (NS+HIR), LPS injection followed by HI (LPS+HI), and LPS injection followed by HI/reperfusion (LPS+HIR) groups. **(A, B)** Immunohistochemical staining on P5 showed significantly increased numbers of Iba-1-positive amoeboid microglia in the LPS+HI and LPS+HIR groups compared to the other groups. **(C, D)** Nissl stain on P16 showed more brain area loss (calculated as [1-ipsilateral/contralateral cortical area] x 100%) in the ipsilateral hemisphere of the LPS+HIR group compared to that of the NS group. Immunohistochemical staining on P16 demonstrated substantially decreased MBP **(E, F)** and increased GFAP **(G, H)** immunodensity in the LPS+HI and LPS+HIR groups compared with the other groups. ★The immunohistochemical pictures in (A, E, G) were photographed from the ipsilateral hemisphere marked with a blue square. $^{\$}p<0.05$ for LPS+HI vs. NS, LPS, NS+HI and NS+HIR; $^{\#}p<0.05$ for LPS+HIR vs. NS, LPS, NS+HI and NS+HIR; $^{*}p<0.05$ for LPS+HIR vs. NS; $^{\&}p<0.05$ for LPS+HI vs. NS, LPS and NS+HI. Iba-1, ionized calcium binding adaptor molecule 1; MBP, myelin basic protein; GFAP, glial fibrillary acidic protein.

Supplementary Fig. 2

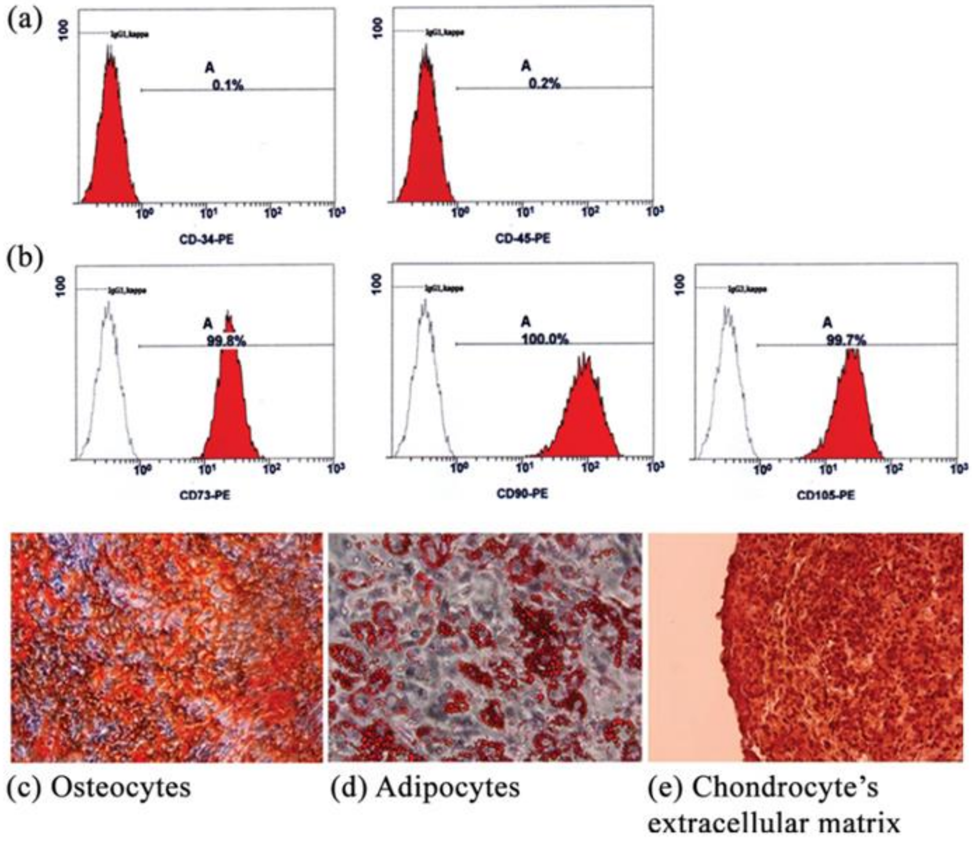

Legend. Mesenchymal stem cell (MSC) identification and differentiation ability determination. MSCs were identified by negative markers for CD34 and CD45 **(a)**, and positive phenotypic markers for CD73, CD90 and CD105 **(b)** using flow cytometry. MSCs were further verified by their ability of differentiation into the osteogenic lineage, shown by Alizarin Red stain **(c)**, the adipogenic lineage, shown by Oil Red O stain **(d)**, and the chondrogenic lineage, shown by Safranin O stain **(e)**.

Supplementary Fig. 3

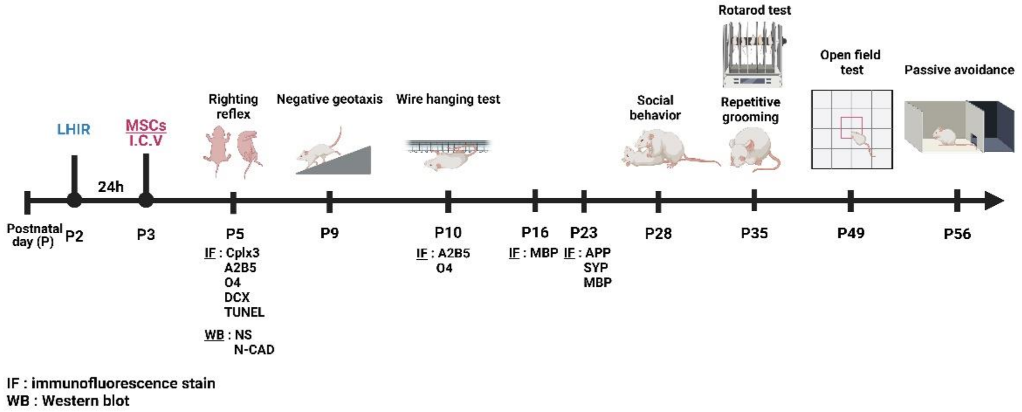

Legend. Experimental protocol. Postnatal day 2 (P2) rat pups were randomly assigned to three groups – sham control (without lipopolysaccharide [LPS], hypoxic ischemia [HI], and reperfusion), LPS-sensitized HI with reperfusion (LHIR), and LHIR followed by intracerebroventricular (i.c.v) infusion of mesenchymal stem cells (MSCs) at 24 hours after LHIR (P3). Immunofluorescence stain (IF), TUNEL stain and Western blotting (WB) were performed at one or more time points between P5 and P23. The neurological tests for cognitive, motor and behavioral function were assessed at several time points between P5 and P56. APP, amyloid precursor protein; Cplx3, complexin-3; DCX, doublecortin; MBP, myelin basic protein; NS, neuroserpin; N-CAD, N-cadherin; SYP, synaptophysin.

Supplementary Fig. 4

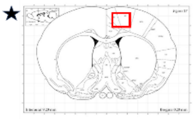

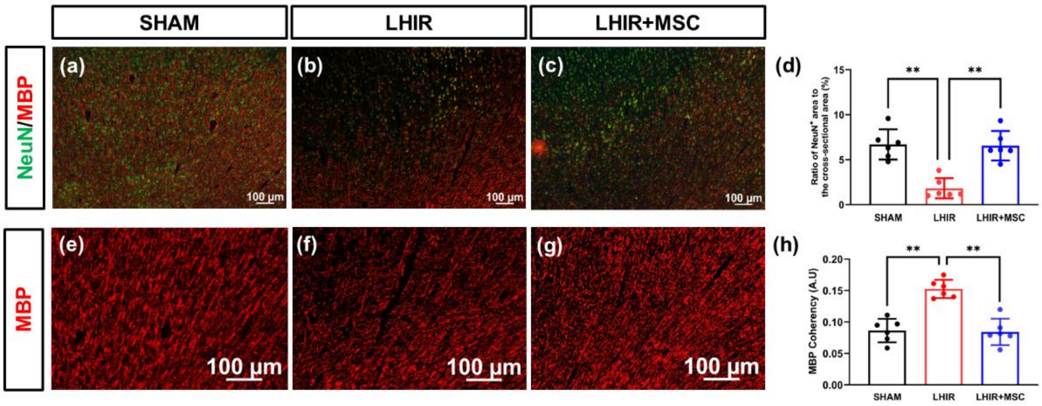

Legend. Immunofluorescent staining of neurons and myelinated axons in the contralateral hemisphere on P23 after LHIR injury. **(a-d)** The LHIR group had significantly decreased cell numbers of NeuN-positive neurons compared to the sham and LHIR+MSC groups. **(e-h)** There was substantially increased coherency of MBP-positive myelinated axons in the LHIR group than in the sham and LHIR+MSC groups, while the latter two groups had comparable axonal complexity. ⋆The pictures in (a-c) and (e-g) were photographed from the contralateral hemisphere marked with a red square. n = 12 in each group. Values are mean ± SD. $^{**}p<0.01$. LHIR, lipopolysaccharide-sensitized hypoxic ischemia/reperfusion; MSC, mesenchymal stem cells; MBP, myelin basic protein.

Supplementary Fig. 5

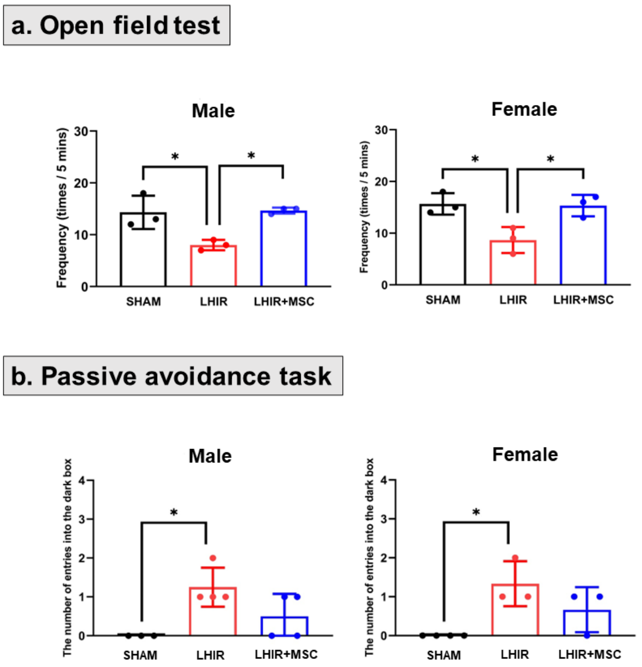

Legend. Behavioral performance and cognitive function in male and female rats at the ages corresponding to sexual maturity. **(a)** Open field test on P49 showed significantly less exploratory behavior in the LHIR group than in the sham and LHIR+MSC groups. There were no sex-related differences in the frequencies. **(b)** Passive avoidance task on P56 demonstrated rats in the LHIR group had erroneous entry into the dark box more frequently than did those in the sham group, while MSC treatment reduced the frequency. The male and female rats had similar performance. n = 4 in each group of male or female rats. Values are mean ± SD. *p<0.05. LHIR, lipopolysaccharide-sensitized hypoxic ischemia/reperfusion; MSC, mesenchymal stem cells.

Supplementary Fig. 6

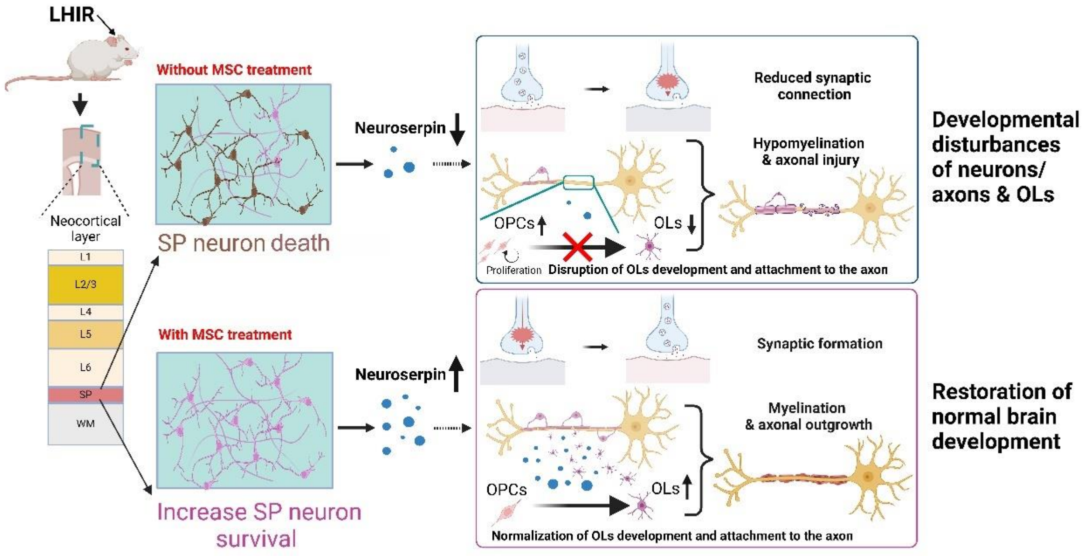

Legend. A proposed diagram illustrating the potential roles of neuroserpin in the reparative mechanisms of mesenchymal stem cell (MSC) therapy after LPS-sensitized hypoxic-ischemia/reperfusion (LHIR) brain injury. **Upper row:** LHIR injury triggered cell death and lowered neuroserpin production in subplate (SP) neurons, leading to the developmental disturbances of neurons/axons and oligodendrocytes in the immature brain. **Lower row:** MSC therapy might alleviate the developmental disturbances through facilitating survival of subplate neurons with restoration of neuroserpin synthesis. OLs, oligodendrocytes; OPCs, oligodendrocyte precursor cells.
